# The Great Amalgam Fallacy

**Published:** 1904-01

**Authors:** Rodrigues Ottolengui

**Affiliations:** New York


### THE GREAT AMALGAM FALLACY.[1]

BY RODRIGUES OTTOLENGUI, M.D.S., NEW YORK.

At the meeting of the New Jersey State Dental Society, last summer, I presented a paper relative to the filling of children's teeth. In the discussion that ensued I was told by several gentlemen that I was threshing old straw. In reply I pointed out that the chief idea exploited—namely, the filling of children's sixth-year molars with gold, rather than with amalgam—was a theory of practice which I had not seen previously advocated, and I asked those who called my paper "old straw" to indicate where in our literature I could find previously printed papers on the same lines. This was not done then, nor has it been done since, notwithstanding the fact that since that time I have read two more papers on the same subject, one before the new National Association of Canada and one before the Second District Society at Brooklyn. Should similar contention be made in regard to the present paper, it will

---

[1] Read before the Academy of Stomatology, Philadelphia, April 28, 1903.

be incumbent upon the disputants to specify the exact features which are so old as to be beyond further discussion.

In the papers mentioned I decried the use of amalgam in children's teeth, and more especially in cavities which are in the initial stages of decay. One result is that I have obtained something of a reputation as an antagonist of amalgam in general, which impression is erroneous.

In this paper, therefore, undertaking a discussion of amalgam within a realm where the highest attainments in the use of the material should maintain, since the most prominent disciples here abide, I shall hope for a sufficient close attention, so that, in the discussion, what I do say and do mean will receive criticism, rather than that there may be any further mistaken ideas in regard to those theories of practice which I advocate.

A word about the title which I have selected. What is "the great amalgam fallacy"? I take it that the general use of amalgam, as it is employed by three-fourths of the dentists of this country, is fallacious in many vital respects, which I shall endeavor to point out in detail. Of the other fourth of all the dentists, a large percentage use amalgam injudiciously and improperly, in at least some of their operations. Indeed, I venture to believe that not ten per cent. of all the amalgam fillings made are properly inserted—that is to say, placed where they will prove most reliable—and the operations performed in a skilful manner throughout.

The chief fallacy in regard to amalgam, and the one which is the basis of all other errors in connection with its use, is that it is a *cheap* filling. This is a more dangerous fallacy, because it rests upon a modicum of truth. In a large share of cases the amalgam filling will occupy less time than would the insertion of a gold filling in the same situation, and time being the chief measure upon which to base the size of a fee, the fee for amalgam is properly smaller than for gold. But in two important classes of cavities this is not true. In very simple cavities the actual time occupied in placing the gold and finishing the same will be no greater than that required for placing the amalgam, plus the time required at a second sitting for polishing, for on that occasion the dentist must charge to profit and loss all the time from the admission to the discharge of the patient. The exception would be where other work were in progress, but evidently the average dentist begrudges even this minimum expenditure of time, if I may judge by the very small number of amalgam fillings that are polished; and here I mean polished with the same care and completeness as the same dentist would bestow upon the same tooth were it filled with gold.

The second and more important class of cavities are those of large size where the work may be complicated by the difficulties of the environment. I allude particularly to cavities which involve the approximal surfaces and which lie close to or beneath the gum margins. The examination of a hundred teeth with cavities of this character filled with amalgam, removed from the mouth, and compared with a hundred similar teeth filled with gold, will show how inadequate is the usual polishing of such large amalgam fillings. Why? Because the time required in sittings subsequent to that at which the amalgam is placed, for the separation of the teeth and proper polishing, is time lost to the dentist unless his fee is as large as for gold, and he has mistakenly educated his patients up to the idea that amalgam is a cheap substitute.

Let us finish the consideration of this aspect of the case before proceeding farther. There are two methods in vogue for the regulation of dental fees, neither of which alone is adequate. One of these, perhaps the one most used, is the fee system. In this course of business management the practitioner erects a definite, if somewhat elastic, scale of prices. "Small gold fillings, ten cents; medium, fifteen to twenty-five; large, thirty to fifty. Amalgam fillings at half the cost of gold." In many of the smaller towns, even respectable, ethically inclined dentists do not hesitate to print a "scale of prices" on the back of their appointment cards. This system makes of a dentist little more than a shopkeeper, a man with wares to sell, rather than personal services to render for adequate return. The arrangement of fees in accordance with the size of a filling may often deprive the dentist of his merited reward for skilled service, and just in proportion as this becomes true in his practice, will such a man become less skilful. It is exactly in this way that the fee system operates against the best results with amalgam. The low price having been established, the dentist discovers that literally he cannot afford to give his amalgam fillings the same thorough attention as he would devote to gold.

The other system of charges is based upon the time required in performing an operation. The dentist computes the time and charges at a definite rate per hour. Thus he classes himself with the day laborers, the bricklayers, plasterers, and other mechanics. He might well organize a union, and have a fixed scale, with strikes when the community demurs at his charges. This man also finds himself underpaid for his amalgam work, because he does so quickly the actual filling, and because he lacks the courage to include in his bill (or records) a separate item of charge for the time occupied in polishing. Thus by both fallacious methods of charging the amalgam work, being the least remunerative, receives less skill, and I do not hesitate to admit that much of the failure with amalgam is more rightfully chargeable to faulty manipulation rather than to the material *per se.*

How may this be remedied? It appears to me that there is another way of arranging fees; one that is more professional, and which will prove more just to patients and more uniformly satisfactory to the dentist. The charge should be proportionate to the service rendered. The material used, the size of the fillings, and the time occupied may all be taken into consideration in deciding the value of the service, but should never be the sole basis of calculating the size of a bill. For example, let us imagine a difficult cavity in a lower third molar, disto-approximal and morsal surfaces involved. Perhaps, for the sake of the higher fee, a band matrix may be placed in position, napkins adjusted and held with clamps, a saliva injector used, and a gold filling inserted, large hunks of some plastic gold being pushed to place with heavy pluggers, the dentist the while struggling to finish before the tide rises to the point of flood. The same tooth might be filled, with less trouble to the patient as well as operator, with amalgam, and of the two operations the amalgam filling may prove the more durable, because in the environment it was more possible to make a perfect operation with amalgam than with gold. Why, then, should the dentist feel that he is entitled to a larger fee for the gold operation, which in his inner consciousness he knows is not up to even his own standards, merely because the material was of gold, and the operation occupied a longer time? Or, to read the same problem reversely, why should he receive a smaller fee for a perfect operation with amalgam than for an imperfect gold filling?

A joke will sometimes point a moral more swiftly than more elaborate language, and you all have heard of the Irishman who thought his dentist overcharged him for extracting a tooth, because another dentist had taken ten times as long and hurt him a great deal more for the same amount of money on a previous occasion. It is almost as bad to believe that we earn a larger fee by prolonging the time our patients spend in the chair.

I am insistent on this subject, because I truly believe that the tremendous fallacy of basing charges upon the material used in filling teeth has deprived dentistry of the best possible services obtainable with amalgam.

It may seem bad taste to inject the personal aspect into this discussion, but I do so to prove the possibility of my contention. I have for a number of years managed my own business along the lines indicated, charging neither by time, fixed fees, nor for materials used. The result is that I have had all classes of patients, rich and poor, and that from any of them I can obtain a higher price for some amalgam fillings than for many gold fillings. It is a significant fact that the "dental parlor" men, advertising amalgam fillings at fifty cents, can persuade a patient to have a living tooth, with moderate sized cavity, covered with a gold cap, paying five or ten dollars for this abominably bad service. Why, then, cannot the ethical dentist receive as large a fee for properly filling the tooth, rendering a vastly superior service for a fee that will remunerate him for the time and skill expended, even though amalgam be used? Evidently it is a matter of education, and heretofore patients have been improperly educated in this regard, and the dentist has none but himself to thank or blame for his present predicament.

Another serious fallacy in relation to amalgam is that the word amalgam is applied indiscriminately to hundreds of totally different preparations. When we speak of a gold filling, we always allude to a filling made of a metal of a definite fineness. The differences in the various kinds of gold are not very material to the resultant fillings, except, perhaps, as there may be a difference between the old style soft gold and the present day cohesive gold, or between the foils and the plastics. But these differences are well defined and fully taken into consideration in the technique of the operations. This is not true of amalgam. All sorts of alloys are similarly used, and a single alloy may be used in many different ways. What wonder that the results with amalgam have been so diverse. The remedy, as applied to the whole profession, cannot perhaps be found within a brief period. The day when all dentists will use the same alloy, in the same manner, and in the same class of cavities is far off. The remedy for any one practitioner is, however, within his own immediate grasp. Let him select one alloy and use no other, whatever the temptation. In making such a choice, if the dentist be at the outset of his career, I would recommend some alloy that has been continuously on sale for a number of years, rather than any of the newer products. I do not mean that these later-day preparations may not be good, but the older ones would not be still on sale had they not been proved to have some value. Having made a choice, the dentist may soon so familiarize himself with this one alloy as to be able to get the very best possible service from it.

A third fallacy relates to cavity preparation, and is one of the evils following in the rear of the notion that amalgam fillings must be cheap. I take it as sound doctrine that cavity preparation, aside from mere shaping for retention, has no relation to the filling-material which is to be inserted. If it can be shown that the preservation of the tooth from recurrence of decay depends upon a definite extension, such as the removal of infected as well as carious dentine, the trimming away of ragged enamel, the arrangement, shape, and placing of the enamel margins and the polishing of the same, then thorough, honest practice demands the same careful cavity preparation for amalgam as for gold. Indeed, if anything, more care is demanded, since it is generally believed that amalgam suffers an alteration of shape. I know that this will be subject for dispute, and that some will argue that the preparation of the enamel margins alone will be more tedious for gold than for amalgam. But why? Any amount of marginal preparation for gold must be based upon the idea that such shaping will render the tooth safer from recurrence of decay after filling than had this precaution not been taken. It may also be true that identical preparation cannot be made with amalgam. For example, many advocate a bevelling of all margins and overlapping of the same with gold. This cannot be done with amalgam, as, when attempted, the amalgam chips away and invites disaster. But because this is true, it is no good evidence that less careful preparation of the margins should be made for amalgam than for gold, and I am convinced that the best results with amalgam are only attainable where the margins are properly and definitely shaped. Having admitted that one should not bevel edges where amalgam is to be used, it follows logically that all bevelled edges must be avoided and those already bevelled should be altered. I believe that there is no amalgam that has strength to resist the stress of mastication if brought to a feather edge. Consequently all margins should meet the outer surface of the tooth in a strong, but well defined angle, without bevelling. Moreover, the absolute edge should be smooth. Cavity preparations, therefore, may be slightly different for amalgam than for gold, but it is equally requisite that it should be carefully, skilfully and thoroughly done. It rarely is.

A fallacy of some importance, I think, has occurred in the work of our eminent men who have carried on tests for this material. The majority of these tests have been made in matrices of glass or steel, and while much valuable knowledge has been attained in regard to the physical properties of various alloys, and of these alloys when amalgamated, I know of no one who has made tests to give us definite knowledge in relation to the best mode of shaping cavities for the reception of amalgam. I venture to suggest that those who have not considered this matter as one of importance will personally try the experiments which I have made and will now report.

Let me cite, as leading up to the purpose of the experiments, a difficult yet common class of cavities found in every-day practice, and more often filled with amalgam than with gold. Imagine a case where the sixth-year molar has a large disto-approximal cavity, while the adjacent twelfth-year molar has a similarly large mesio-approximal cavity, both involving the morsal surfaces. Preparation of these cavities, so far as removal of carious matter is concerned, discloses the fact that both pulps may be saved alive, but that in both instances the gingival margins must lie at or under the gum. Of course, every gentleman present makes it a practice to properly fill these teeth, even when using amalgam. But let us discuss the work of the men who are not present,—those who get small and stated fees for amalgam.

Different procedures maintain. Some fill both teeth at the same sitting. Of these, a great many polish only the visible and consequently accessible parts of the filling at the second sitting. Still fewer pass a narrow strip of sand-paper along the gingival margins, and polish them in that way, the moisture, blood, and débris, of course, being no hinderance. Another few may next use a steel separator and obtain enough space to polish the contact points, in which case, even though the fillings be polished, they lose their contact.

Following another method, we have those who fill one cavity at the first sitting and polish it at the next, before filling the second. This is better, but the same dilemma maintains when the polishing of the last filling is attempted. Either the contact point remains unpolished, or else it is lost.

Of course, the proper method is familiar to all. The teeth must be wedged apart,—one sitting; one tooth filled,—two sittings; this one polished and the other filled,—three sittings; the second filling polished and the teeth allowed to fall back again,—four sittings. But even with amalgam this kind of work cannot be done for small fees, and that is why it is done so seldom.

I now with some hesitation present a new method of procedure. I do this tentatively only, not giving it a final endorsement, because I have not yet sufficiently tested it. I will say, however, that it seems to promise a satisfactory solution of this difficult problem, and that it allows the filling of the teeth in two instead of four sittings. The cavities are prepared, with the dam in place, of course, the same as for a porcelain inlay, all edges being made sound, smooth, and well defined with small stones. I then make a gold matrix for one cavity, and with the matrix in place fill the tooth with a quick-setting amalgam, building out the approximal contour slightly to an excess. The matrix is then removed with the filling, which is easily done, and of course the matrix suffers no alteration in shape. This is set aside for the time being. The second cavity is similarly treated, and the two are then filled with gutta-percha and the patient dismissed, which ends the first sitting. Each matrix with its contained amalgam filling is then dropped upon a small pile of freshly mixed plaster, which is made to invest the matrices, and then trimmed to satisfactory form for handling. On the following day it will be found that the amalgam can be trimmed to shape and polish, the gold serving as a guide to the true cavity margins. It is best to leave the polishing of the morsal surfaces until after setting the inlays. It is manifest that by this means the gingival margins are easily polished. Some excess is left at the contact points until the patient presents, at which time the dam is again placed and the final polishing and fitting achieved, after which each is separately set as an inlay, and when completed the two amalgam fillings have perfect polish and perfect margins, regardless of the difficulties which would ordinarily be met; moreover, the contact can be made absolute; indeed, the two fillings may be literally wedged between the teeth.

Even if this work is not done in the mouth, it will be most instructive to try a few such fillings out of the mouth. The opportunity to handle the filling, polish it, and replace it in the cavity from time to time for examination will prove more convincing than endless argument that amalgam overlapping a margin has no strength. A thin edge anywhere will be seen to crumble and break under the action of even the finest cuttle-fish disks, and this, more than anything, will satisfy the most sceptical that a definite marginal shape is essential with amalgam. It will also lead him to the thought that the trenches about old fillings which so long have been attributed to *shrinkage,* may in a large percentage of cases more properly be explained by *breakage,* due to overlapping the margins.

One other procedure I will suggest for future trial by the profession. In masticating, or, indeed, in other surfaces where it is desired to retain the filling in the usual way, by retentive shaping, the matrix may still be used, and I think with advantage. In such cases the matrix is first gently pressed into the cavity, getting it as deep as possible without breaking the gold, and then perfectly burnishing to the margins. Next, the gold may be pressed tightly against the floor and walls with spunk, permitting it to break where it will. This restores the retentive shape of the cavity, which is now lined with gold. Into this, without removing the matrix, the amalgam is placed and the excess of gold subsequently torn away. This will give an amalgam filling, but the cavity walls will be lined with gold, which will, I think, permanently prevent discoloration of the dentine by oxidation. Contrary to what might be supposed, the amalgam does not unite with the gold, and the mercury does not penetrate it during setting. I cannot see why it should do so later.

A slight variation of the method will appeal to those who like a cement joint. The matrix being made, but not burnished sufficiently to break it, a thin cement is smeared over all the walls, and the matrix put in the cavity and packed tight with spunk, the excess of cement being carefully forced outward under the matrix and over the margins. After this hardens, the amalgam may be inserted at the same sitting.

There are many other fallacies in regard to amalgam and the methods of its usage, but I believe I have pointed out the more mischievous ones.

